# Investigation of mental health among hospital workers in the COVID-19 pandemic: a cross-sectional study

**DOI:** 10.1590/1516-3180.2020.0272.R3.21072020

**Published:** 2020-10-09

**Authors:** Songül Araç, Süleyman Dönmezdil

**Affiliations:** I MD. Assistant Professor, Department of Emergency Medicine, University of Health Sciences, Diyarbakır Gazi Yaşargil Eğitim ve Araştırma Hastanesi, Diyarbakır, Turkey.; II MD. Assistant Professor, Department of Psychiatry, University of Health Sciences, Diyarbakır Gazi Yaşargil Eğitim ve Araştırma Hastanesi, Diyarbakır, Turkey.

**Keywords:** Anxiety, COVID-19 virus disease, Depression, Pandemics, Health worker, Sleep disorder, Quality of life

## Abstract

**BACKGROUND:**

The rapid spread of the COVID-19 epidemic has led to extraordinary measures taken worldwide, and has led to serious psychological disorders. Healthcare professionals face greater severity of stress burden, due both to their direct contact with patients with the virus and to the isolation dimension of this outbreak.

**OBJECTIVE:**

To examine psychiatric disorders such as anxiety, depression and sleep disorders among healthcare professionals working in an emergency department and a COVID-19 clinic.

**DESIGN AND SETTING:**

Cross-sectional study including healthcare professionals in the emergency department and other units serving patients with COVID-19, of a training and research hospital in Turkey.

**METHODS:**

210 volunteers, including 105 healthcare professionals in the emergency department and 105 healthcare professionals working in other departments rendering services for COVID-19 patients, were included in this study. A sociodemographic data form and the Hospital Anxiety Depression Scale (HAD), Pittsburg Sleep Quality Index (PSQI), World Health Organization Quality of Life scale (WHOQOL-BREF-TR) and Religious Orientation Scale were applied to the volunteers.

**RESULTS:**

The perceived stress levels and PSQI subscores were found to be significantly higher among the volunteers working in the emergency department than among those in other departments. The risk of development of anxiety among women was 16.6 times higher than among men.

**CONCLUSIONS:**

Healthcare professionals on the frontline need systematic regular psychosocial support mechanisms. Anxiety due to fear of infecting family members can be prevented through precautions such as isolation. However, it should be remembered that loneliness and feelings of missing family members consequent to isolation may increase the risk of depression.

## INTRODUCTION

A new virus that emerged in Wuhan, China, in 2019 has been found to spread quickly and cause pneumonia, leading to severe respiratory failure. It was named SARS-CoV-2 due to its close resemblance to SARS-CoV, and the disease was named COVID-19. The virus spread rapidly across China and has become a significant health problem worldwide. Considering the rate of spreading of the virus, COVID-19 was declared to be a pandemic by the World Health Organization (WHO) on March 12, 2020. As of April 30, 2020, the number of cases had exceeded 118,000 in Turkey and 3,190,000 worldwide. Although mortality rates vary from country to country and according to the number of tests performed, the mortality rate in Turkey is approximately 2.33%.^1^^,^^2^

Upon the announcement declaring COVID-19 to be a pandemic, measures were quickly implemented in Turkey and worldwide. Some of the warnings issued by the authorities related to anxiety and post-traumatic stress disorder that may arise in the community, in connection with exposure to the virus.^3^ The rapid spread of the epidemic in countries such as Italy and Spain, and the disruptions to the healthcare systems of these countries, led to rapid organization of the healthcare system in Turkey. COVID-19 hospitals were designated and planning was undertaken with regard to patient admission, diagnosis, treatment protocols and work systems for the healthcare staff employed in these locations.

In addition to these measures, numerous meetings were held and training sessions were organized with the aim of informing healthcare professionals about the virus in Turkey, as was done in many countries with the outbreak. However, because there are many unknowns about the virus and because healthcare professionals have to perform triage for treatments in countries where the outbreak is very severe, the anxiety levels among healthcare professionals have risen.^4^^,^^5^ During this challenging process, these high levels of anxiety among healthcare professionals will naturally be neglected and will not receive the necessary attention. Nonetheless, this issue should not be ignored, since neglect now may lead to problems that are difficult to solve later on.

Many psychiatric disorders may occur in situations of disease outbreaks or natural disasters. Among these disorders, anxiety, depression and post-traumatic stress disorder are the most common. A study conducted in China among healthcare workers involved in the COVID-19 outbreak supports this idea. In that study, Kang et al.^4^ found that anxiety, depression and sleep disorder scale scores were significantly higher among physicians and nurses.

Unless corrected through adequate psychological support mechanisms, emergence of psychiatric disorders can lead to deterioration and impaired functioning. As shown in many studies, intense stress and anxiety lead to weakness in the immune system and can lead to healthcare professionals becoming infected quickly during the outbreak.^6^^,^^7^

Protecting the mental health of healthcare workers involved in the pandemic is important. Through this, mental disorders may be prevented before they occur. Development of protective psychosocial support mechanisms through advance knowledge of the risk groups among healthcare workers is essential for the protection of public health.

Emergency department workers serve patients who have not yet been diagnosed. In addition, they have more contact with patients in terms of diagnosis and treatment. Workers in COVID-19 clinics serve diagnosed patients and their contact with patient is more limited than that of emergency workers. Therefore, there is a need to investigate the difference in anxiety situation between these two groups of workers.

## OBJECTIVE

The aim of this study was to examine psychiatric disorders such as anxiety, depression and sleep disorder among healthcare professionals working in an emergency department and in a COVID-19 clinic.

## METHODS

This research was started after approval had been obtained from the clinical research ethics committee of the training and research hospital of a local health sciences university (date: March 13, 2020; number: 449).

The study subjects were healthcare professionals in the emergency department and other units serving patients with COVID-19, in a training and research hospital in Turkey. The effect of interventions made among COVID-19 patients with exact diagnoses was assessed in relation to two groups of healthcare professionals. The primary group consisted of the emergency department medical team, which performs interventions without knowing their patients’ diagnoses. The secondary group consisted of the healthcare team that provides care for hospitalized patients whose clinical, imaging and serological diagnoses have been made by infectious disease and chest disease specialists.

Our intention was to reach all employees in the emergency department. Therefore, all emergency service workers who agreed to participate in the study were included. The comparison group was selected from among employees in the COVID-19 clinic who presented similar sociodemographic characteristics. After forming the groups, 210 volunteers (105 primary and 105 secondary group employees) were included in the study after their consent had been received. The participants included physicians, nurses, data-entry staff, patient transportation staff, and patient support staff. At the time of forming the groups, these professionals were divided into three groups: physicians, nurses and other medical staff. Twelve participants were found not to have filled out the questionnaires appropriately and were excluded from the research. Thus, data from 198 volunteers were included in the study.

A sociodemographic data form and the Hospital Anxiety Depression Scale (HADS), Pittsburgh Sleep Quality Index (PSQI), World Health Organization Quality Of Life scale (WHOQOL-BREF-TR) and Religious Orientation Scale were applied to the volunteers.

**Hospital Anxiety and Depression Scale (HADS):** This scale, developed by Zigmond and Snaith^8^ contains a total of 14 items. Seven questions on the scale assess anxiety and seven assess depression. The scale consists of four-point Likert-type questions, which are filled out by the individuals surveyed. On both the anxiety and the depression subscales, a score of 11 and above indicates a severe condition.

**Pittsburgh Sleep Quality Index (PSQI):** This scale, developed by Buysse et al.^9^ in 1989, is the most widely used scale among sleep disorder-specific scales. Seven subscales are evaluated, with a total of 18 questions in the complete PSQI. These seven subscales relate to subjective sleep quality, sleep latency, sleep duration, habitual sleep efficiency, sleep disturbances, use of sleeping medication and daytime sleep dysfunction. The response to each item is scored from 0 to 3, according to the frequency of symptoms. Higher values indicate poorer quality of sleep and higher levels of sleep disturbances. In this study, all the subscales of the PSQI were addressed.

**World Health Organization Quality of Life scale (WHOQOL-BREF-TR):** This was developed by the World Health Organization (WHO) for subjective evaluation of quality of life.^10^ The aim of the scale items is to investigate the quality of life in five domains. These five domains relate to physical, mental, social, environmental and general health. Higher scores indicate higher quality of life.

**Religious Orientation Scale:** The religious orientation scale (ROS), developed by Allport and Ross, is composed of five-point Likert-type items.^11^ The scale is divided into three subscales: intrinsic religious orientation, extrinsic-personal religious orientation and extrinsic-social religious orientation. Higher scores on the scale indicate greater significance of the relevant subscale. A high score from a given subgroup means that belief within that subgroup (for example, internal religious orientation) is high.

### Statistical evaluation

The data were analyzed using the SPSS 16.0 software package (SPSS Inc, Chicago, United States). Analysis on the categorical data was performed using the chi-square test. Student's t test was used in binary groups of analysis on quantitative data. Covariance analysis was applied to the variables, which were deemed statistically significant according to the results from the logistic regression analysis.

Logistic regression analysis was performed to determine the risk factors for severe development of anxiety and depression. Firstly, single-variable logistic regression analysis was performed for all the data obtained in the study. Following this, the variables with P < 0.05 were subjected to multivariate logistic regression analysis, as covariance factors. A statistical significance level of P < 0.05 was used in all analyses.

## RESULTS

In the analysis on the sociodemographic data, there were no statistically significant differences between the groups (Table 1).

**Table 1:** Comparison of categorical and numerical sociodemographic data between the groups

		Emergency team (n = 100)n (%) or mean ± SD	Other teams (n = 98) n (%) or mean ± SD	χ^2^	P
**Gender**					
	Female	42 (53.8)	36 (46.2)	0.448	0.2
	Male	58 (48.3)	62 (51.7)		
**Marital status**					
	Single	28 (43.8)	36 (56.3)	0.189	0.2
	Married	72 (53.7)	62 (46.3)		
**Profession**					
	Physician	34 (53.1)	30 (46.9)	4.53	0.1
	Nurse	29 (40.8)	42 (59.2)		
	Other	37 (58.7)	26 (41.3)		
**Living arrangements**					
	Living with parent	24 (48.0)	26 (52.0)	1.262	0.7
	Living with spouse and children	66 (53.2)	58 (46.8)		
	Sharing a bachelor apartment	4 (40.0)	6 (60.0)		
	Living alone	6 (42.9)	8 (57.1)		
**Chronic disease**					
	Yes	14 (45.2)	17 (54.8)	0.517	0.3
	No	86 (51.5)	81 (48.5)		
**Diagnosis of the disease**					
	**Hypertension**	5 (45.5)	6 (54.5)	0.517	0.9
	**Diabetes mellitus**	1 (33.3)	2 (33.3)		
	**Other**	9 (52.9)	8 (47.1)		
**HADS severity of anxiety**					
	Marked severity	60 (60.0)	18 (18.4)	35.93	< 0.001
	Borderline or normal	40 (40.0)	80 (81.6)		
**HADS severity of depression**					
	Marked severity	51 (51.0)	11 (11.2)	36.40	< 0.001
	Borderline or normal	49 (49.0)	87 (88.8)		
**Age (years)**		35.2 ± 6.4	33.71 ± 7.2		0.1
**Number of children**		1.34 ± 1.2	1.04 ± 1.05		0.07
**Length of education (years)**		14.05 ± 2.9	14.38 ± 2.7		0.4
**Professional experience (years)**		10.2 ± 6.5	9.4 ± 6.9		0.4
**Length of time working in the unit (years)**		4.8 ± 4.2	4.6 ± 4.5		0.7

HADS = Hospital Anxiety and Depression Scale; HADS anxiety of marked severity = anxiety subscale score of 11 and above on HADS; HADS depression of marked severity = depression subscale score of 11 and above on HADS; SD = standard deviation.

Among the HADS scores, it was noteworthy that a high number of the participants scored 11 and above, which was indicative of the presence of severe anxiety and depression. The anxiety and depression subscale scores were above 11 points for 39.4% and 31.3% of these individuals, respectively. From the perspective of primary and secondary encounters with potential COVID-19 patients, it was found that the scores on the anxiety and depression subscales were both significantly higher in the group that was facing potential COVID-19 cases first (P < 0.001; Table 1).

It was observed that both the anxiety and depression HADS scores were significantly higher among emergency staff (Table 2).

**Table 2:** Statistical analysis on the scores for the scales that were applied to the groups

	Emergency team (n = 100) Mean ± SD	Other teams (n = 98) Mean ± SD
Religious orientation − intrinsic orientation	41.2 ± 6.7	40 ± 6.3
Religious orientation − external personal orientation	24.2 ± 5.3	23.9 ± 5.1
Religious orientation − social religious orientation	13.7 ± 4.4	12.7 ± 4.5
HADS anxiety score**	12 ± 4.5	7 ± 3.4
HADS depression score**	10.8 ± 4.4	6.4 ± 3
PSQI subjective sleep quality**	1.58 ± 0.8	1.1 ± 0.6
PSQI time to fall asleep**	1.8 ± 0.9	1.1 ± 0.9
PSQI sleep duration*	1.1 ± 1	0.7 ± 0.8
PSQI habitual sleep efficiency	0.5 ± 0.9	0.3 ± 0.7
PSQI sleep disturbances	1.5 ± 0.7	1.4 ± 0.9
PSQI use of sleep medication**	0.2 ± 0.5	0.4 ± 0.1
PSQI daytime dysfunction **	1.47 ± 0.9	0.6 ± 0.7
Perceived stress level score**	28 ± 10.6	17.7 ± 7.1
WHOQOL overall health**	5.5 ± 1.5	6.4 ± 1.7
WHOQOL physical health**	22.2 ± 4.6	25.5 ± 4.7
WHOQOL psychological health**	19.3 ± 4.2	21.8 ± 3.7
WHOQOL social relation**	9.5 ± 2.2	11.2 ± 3.3
WHOQOL environmental health**	22.9 ± 5.3	25.6 ± 5.4

SD = standard deviation; HADS: Hospital Anxiety and Depression Scale; PSQI = Pittsburgh Sleep Quality Index; WHOQOL: World Health Organization Quality Of Life scale;

* = P < 0.01;

** = P < 0.001.

The perceived stress levels and PSQI subscale scores of the participants in the primary group were significantly higher than those of participants in the secondary group (P < 0.001, Table 2).

The risk of developing anxiety in the female gender was found to be 16.6 times greater than in males. In addition, the relative risk for anxiety development was 8.7 times higher in physicians and 4.8 times higher in nurses when compared with other professional groups (Table 3).

**Table 3:** Statistical differences in the scores for the scales, according to profession

		Physician (n = 61) Mean ± SD or n (%)	Nurse (n = 71) Mean ± SD or n (%)	Other (n = 63) Mean ± SD or n (%)	Total n %
**Sex**					
	Female	14 (17.9)	30 (38.5)	34 (43.6)	78 (100)
	Male	50 (41.7)	41 (34.2)	29 (24.2)	120 (100)
Age		34.81 ± 7.66	34.3 ± 6.83	34.43 ± 6.17	
HADS anxiety score***		7.63 ± 3.7	10.1 ± 4.8	10.8 ± 4.8	
HADS depression score***		7.2 ± 4.1	9.1 ± 4.5	9.5 ± 4.1	
Intrinsic religious orientation***		37 ± 6.9	42 ± 6.3	42.7 ± 4.4	
Personal-extrinsic religious orientation***		21.2 ± 5.3	24.3 ± 5.2	26.7 ± 3.4	
Social religious orientation***		10.6 ± 3.5	13.1 ± 4	16 ± 4	
PSQI subjective sleep quality		1.2 ± 0.7	1.3 ± 0.7	1.4 ± 0.8	
PSQI time to fall asleep**		1.1 ± 0.9	1.7 ± 1	1.4 ± 0.9	
PSQI sleep duration		0.7 ± 0.7	0.9 ± 1	1.1 ± 1	
PSQI habitual sleep efficiency**		0.1 ± 0.3	0.4 ± 0.9	0.6 ± 1	
PSQI sleep disturbances***		1.1 ± 0.7	1.6 ± 0.8	1.7 ± 0.7	
PSQI use of sleep medication		0.1 ± 0.5	0.1 ± 0.5	0 ± 0.1	
PSQI daytime dysfunction		0.9 ± 1	1.1 ± 0.9	1.1 ± 0.8	
Perceived stress level score***		18.6 ± 8.3	23.6 ± 10.6	26.5 ± 10.6	
WHOQOL overall health		6.3 ± 1.4	5.7 ± 1.8	5.8 ± 1.6	
WHOQOL physical health*		25.1 ± 3.9	22.7 ± 5.2	24 ± 5.3	
WHOQOL psychological health		21.2 ± 3.8	20.5 ± 4.3	19.8 ± 4.2	
WHOQOL social relation*		10.8 ± 2.1	10.6 ± 3.8	9.6 ± 2.2	
WHOQOL environment***		27.8 ± 3.9	23.3 ± 5.5	21.7 ± 5.2	

SD = standard deviation; HADS = Hospital Anxiety and Depression Scale; PSQI = Pittsburgh Sleep Quality Index; WHOQOL: World Health Organization Quality Of Life scale;

* P < 0.05;

** P < 0.01;

*** P < 0.001.

In the multivariate logistic regression analysis, gender, profession, HADS-depression, the use of sleeping medication subscale score of the PSQI, the perceived stress level and the WHOQOL physical and environmental domain subscale scores were found to be the effective risk factors (Table 4).

**Table 4:** Multivariate logistic regression analysis results

	95% confidence interval		Odds ratio	P
	Lower	Upper		
Gender (female)	0.015	0.237	16.631	**< 0.001**
Profession (other)	1.110	37.142	1	0.147
Profession (physician)	1.370	55.877	8.750	**0.022**
Profession (nurse)	1.036	22.667	4.845	**0.045**
Working unit (primary)	0.660	13.058	1.998	0.158
Intrinsic religious orientation	0.938	1.272	1.290	0.256
Personal-extrinsic religious orientation	0.956	1.376	2.181	0.140
HADS-depression	1.131	1.771	9.194	**0.002**
PSQI subjective sleep quality	0.742	4.560	1.734	0.188
PSQI time to fall asleep	0.911	3.373	2.824	0.093
PSQI sleep duration	0.411	1.783	0.172	0.679
PSQI sleep disturbances	0.411	3.022	0.046	0.831
PSQI use of sleep medication	1.402	14.838	6.357	**0.012**
PSQI daytime dysfunction	0.463	2.452	0.022	0.882
Perceived stress level	1.050	1.278	8.639	**0.003**
WHOQOL overall	0.940	2.962	3.062	0.080
WHOQOL physical	1.072	1.764	6.299	**0.012**
WHOQOL psychological	0.737	1.221	0.167	0.683
WHOQOL social	0.574	1.174	1.165	0.280
WHOQOL environmental	0.640	0.939	6.757	**0.009**

HADS = Hospital Anxiety and Depression Scale; PSQI = Pittsburgh Sleep Quality Index; WHOQOL = World Health Organization Quality Of Life scale.

There were significant differences in the HADS, PSQI and WHOQOL subscale scores between the primary group and the secondary group. On the other hand, while the Religious Orientation Scale score was higher in the primary group, this difference was not statistically significant.

## DISCUSSION

The HADS scores of all the participants in this study showed that a high number of them scored 11 and above, which is indicative of the presence of severe anxiety and depression. The anxiety and depression scale scores were above 11 points for 39.4% and 31.3% of these individuals, respectively. From the perspective of primary and secondary encounters with potential COVID-19 patients, it was found that the scores on the anxiety and depression subscales were both significantly higher in the group that faces potential COVID-19 cases first. This indicates that staff in the primary group, i.e. those involved in the emergency unit, which is the place to which these patients are first admitted, are at higher risk of anxiety and depression. We believe that the higher values for the scores on these scales may have been due to the examinations and interventions that were performed on these patients before their diagnoses had become established. These higher values may also have been due to the working conditions of the emergency department, which are more stressful than those of other work areas.

In a study on emergency physicians, Wong et al. reported that these physicians’ scores on the anxiety and perceived stress scales were higher than those of other physicians.^12^ In the present study, stress levels and stress-related anxiety levels were significantly higher among emergency staff, in line with the literature. In another study, González-Cabrera et al.^13^ compared anxiety and salivary cortisol levels among the emergency service staff on normal days and on shift days. They found that emergency service employees had higher anxiety and salivary cortisol levels. Similarly, the higher levels of anxiety and stress found among the emergency service employees in our study support the hypothesis that changes to cortisol levels may have occurred in the same manner as reported in the previous study. Moreover, this may occur in association with immune system defects.

Stress, which can play a role in the etiology of numerous psychiatric disorders, can be considered to be a symptom of psychiatric disorders. In addition, stress can be both the cause and the result of sleep disorders. The response of the body against stress aims to provide the necessary homeostasis for the survival of life.

Many hormonal and neuronal mechanisms may play a role in this homeostasis. One of these mechanisms is the hypothalamic-pituitary-adrenal (HPA) axis. Sleep quality is among the parameters associated with the layout of the HPA axis.

Cortisol is a stress hormone with significant effects on the immune system. It has the capacity to cause serious immune disorders, depending on its secretion level.^14^ The perceived stress levels and PSQI subscale scores of the members of the primary group in the present study were significantly higher than those of the secondary group.

These high levels of stress and the deterioration of sleep quality among healthcare professionals during the pandemic need to be highlighted. Their immune systems are at a low ebb at these times, which means that these individuals can become infected quickly. If this happens, the healthcare system will suffer loss of functionality.

Studies have shown that attention and decision-making mechanisms are affected in situations of psychiatric disorders, such as anxiety disorder and depressive disorder. Moreover, it has also been reported that stress has negative effects on attention.^15^^,^^16^

Healthcare professionals use their higher cortical functions when making diagnoses and planning treatments. Any mistake made at such times can cause the loss of the patient. Thus, in situations of anxiety, high stress and depression, in which the higher cortical functions are affected, healthcare professionals are more likely to make mistakes.

In order to correct this condition, it is necessary to eliminate the causes that pose the risk. In the present study, HADS-depression, HADS-anxiety and stress level test scores were higher among the primary healthcare professionals than among the secondary healthcare professionals. Considering that increased anxiety, depression and stress levels negatively affects cortical functions, it can be stated that lowering these scores effectively will be a very important factor in preventing transmission of the disease to healthcare professionals. For this reason, systematic support programs for healthcare professionals, including pharmacotherapy options, need to be developed quickly.

Quality of life can be impaired for any reason that affects physical and mental health. In the present study, the negative changes in quality of life detected through WHOQOL were consistent with reports in the literature showing that quality-of-life scale scores were low among patients with high depression scale scores.^17^^,^^18^

The most likely behavior among healthcare professionals, who know that higher burdens of the virus are a negative factor regarding the prognosis, is to keep their contact and communication with their patients at a minimum level, when they meet these patients after admission to the emergency service, for diagnosis and treatment planning. This can be considered to be an instinctive action by professionals to protect themselves. However, it should be noted that fear and anxiety may increase in patients who are already fearful upon admission to the emergency service, as a result of such behavior among healthcare professionals.

If patients are unable to learn the basic information that needs to be learned, such as the diagnosis of the disease, its severity and the duration of the treatment to be administered, they will be more likely to be affected mentally. Conversely, healthcare professionals who do not want to be exposed to the burden of the virus will feel guilty if they fail to inform their patients adequately, given that they will think that they are not adhering to the ethical rules. Healthcare professionals are trapped between, on the one hand, their fear of becoming ill and infecting their family members, which should be seen as normal human behavior; and, on the other hand, the idea that they might not be able to properly inform their patients in an ethical manner. Thus, it should be noted that healthcare professionals constitute an at-risk group for psychiatric disorders in the future.

In the present study, logistic regression analysis was used to determine the risk factors that led to severe HADS scores. Parameters that were found to be significant in univariate logistic regression analysis were included as covariance factors in multivariate logistic regression analysis. Gender, profession, HADS-depression, the use of sleeping medication parameter of the PSQI, perceived stress and the WHOQOL physical and environmental subscale scores were found to be the effective risk factors.

In this study, the risk of developing anxiety among females was found to be 16.6 times greater than among males. In addition, the relative risk of developing anxiety was 8.7 times higher among physicians and 4.8 times higher among nurses than in other professional groups. Higher rates of anxiety and depression among women are an accepted fact.^19^ We believe that these higher rates among women are caused by a pandemic-specific fear of losing a spouse, child or relative, or of infecting relatives with the virus. Among the healthcare professionals, physicians and nurses (who have higher levels of education and experience) were found to have higher risk values. This suggests that physical proximity and longer contact time with COVID-19 patients are more likely to be effective for development of anxiety, rather than the respective knowledge. However, considering the data available in the medical world regarding this disease, it remains true that people in these professions are not knowledgeable enough. Greater knowledge about this disease will decrease the concerns among professionals such as physicians and nurses.

The relative risk of developing anxiety, regardless of the groups, was found to be 9.1 times higher for the HADS-depression variable, 6.3 times higher for the use of sleeping medication parameter of the PSQI, 8.6 times higher for the perceived stress level score and 6.2 and 6.7 times higher for the WHOQOL physical and environmental factors, respectively. Coexistence of depression and anxiety has been the subject of numerous studies.^20^^,^^21^ In cases of higher anxiety, deterioration in sleep quality is an expected risk, especially during an extraordinary period, such as the current pandemic. It can be expected that sleep disturbances for which sleeping medication is required will show scientifically proportionate correlations with the severity of anxiety. A high level of perceived stress may be one of the indicators of high levels of anxiety. There are other studies with the same results in the literature.^22^^,^^23^ In parallel, quality of life is severely affected in all cases of psychiatric or physical disorders, and this is reflected in the scores on scales that measure the quality of life.

In summary, the pandemic has led to anxiety among healthcare workers. This anxiety was found to be higher among females in all three groups (doctors, nurses and other healthcare workers) than among males. There may be multiple reasons for this higher incidence among females. In the general population, high incidence of anxiety disorders among females may be explained by their concerns, as mothers and wives, about infecting relatives with the disease and about the lack of adequate information regarding the course of the disease and future morbidity. The predominance of females among nurses may be the cause of the high level of anxiety within the nursing profession.

### Limitations

This study was limited by its relatively small sample size and low response rate, and because it was conducted at a single academic medical center at one point in time. Therefore, these findings may lack generalizability. Further studies in this field are required, in order to make more confident assessments. This study was also subject to the limitations of the tests that we used.

## CONCLUSION

Healthcare professionals on the frontline need systematic regular psychosocial support mechanisms. Anxiety due to fear of infecting family members can be prevented through precautions such as isolation. However, it should be remembered that loneliness and feelings of missing family members consequent to isolation may increase the risk of depression.

In summary, the pandemic has led to anxiety among healthcare workers. For this reason, systematic support programs for healthcare professionals, including pharmacotherapy options, need to be developed quickly.
